# Infantile undifferentiated sarcomas: a diagnostic and therapeutic challenge – two case reports and literature review

**DOI:** 10.2340/1651-226X.2025.42162

**Published:** 2025-02-10

**Authors:** Aleksandra Stanio, Jakub Czarny, Sandra Rutkowska, Katarzyna Adamczewska-Wawrzynowicz, Łukasz Młynarczyk, Agnieszka Stróżyk, Katarzyna Jończyk-Potoczna, Alicja Bartkowska-Śniatkowska, Jacek Wachowiak, Katarzyna Derwich, Olga Zając-Spychała

**Affiliations:** a Student Scientific Circle, Poznan University of Medical Sciences, Poznań, Poland; b Department of Pediatric Oncology, Hematology and Transplantology, Poznan University of Medical Sciences, Poznań, Poland; c Department of Pediatric Radiology, Poznan University of Medical Sciences, Poznań, Poland; d Department of Pediatric Anaesthesiology and Intensive Therapy, Poznan University of Medical Sciences, Poznań, Poland

**Keywords:** Undifferentiated sarcoma, infancy, chemotherapy, progression, toxicity, refractory

## Abstract

**Background:**

Soft tissue undifferentiated sarcomas (STUS) are an ultra-rare and heterogenous group of mesenchymal neoplasms often lacking known genetic abnormalities with a marked vulnerability towards intensive therapy such as invasive surgery and high dose chemotherapy. Despite aggressive treatment, they are usually associated with dismal outcomes.

**Case presentation:**

Here we describe two cases of STUS in 3-week-old and 3-month-old infants localized on the neck and the trunk area.

**Discussion:**

In both cases, the malignancy had a fatal outcome due to the toxicity of intensive therapy in one case and the progression of the disease in the other. The purpose of this report is to discuss the clinical challenges of managing infancy-related STUS such as limited treatment options and poor prognosis.

## Background

Much has been learned about soft tissue sarcomas in infants over the past few decades. Uniform protocols outlining the care of infants are responsible for the rapid improvement in prognosis for many of these malignancies and have allowed follow-up studies identifying the long-term sequelae of treatment [1]. However, there is still a heterogeneous group of malignancies originating from primitive mesenchymal tissue called soft tissue undifferentiated sarcomas (STUS) that cannot be classified among standardized histopathologic entities. They are characterized by their morphology, genetic alterations, and clinical course [2]. The 2020 World Health Organization (WHO) Classification of Tumors of Soft Tissue and Bone distinguished undifferentiated small round cell sarcomas as a separate group of malignancies which include Ewing sarcoma, round cell sarcoma with EWSR1-non-ETS fusion, CIC-rearranged sarcoma, and sarcoma with BCOR genetic alteration [2]. Compared to other sarcomas, they have poorer prognosis and tend to have a bad response to treatment [3]. The majority of analyses confirm poor outcomes in pediatric, especially infantile, populations [4, 5] with a high recurrence rate in the setting of incomplete surgical resection.

Moreover, STUS can metastasize distantly. Previous publications have largely focused on the pathologic characteristics of this emerging entity. The literature data regarding STUS therapy are limited due to their rarity. Moreover, the approach to intensive treatment during infancy is different than in older children because of the immature physiological system. Given the rarity of the tumor, the unique obstacles to effective local control, and the lack of standard treatment guidelines, herein we present two cases of infantile STUS with fatal outcomes due to toxicity of intensive therapy in one case and due to progression of the disease in the other.

## Case presentation

### Case #1

**Figure 1:**
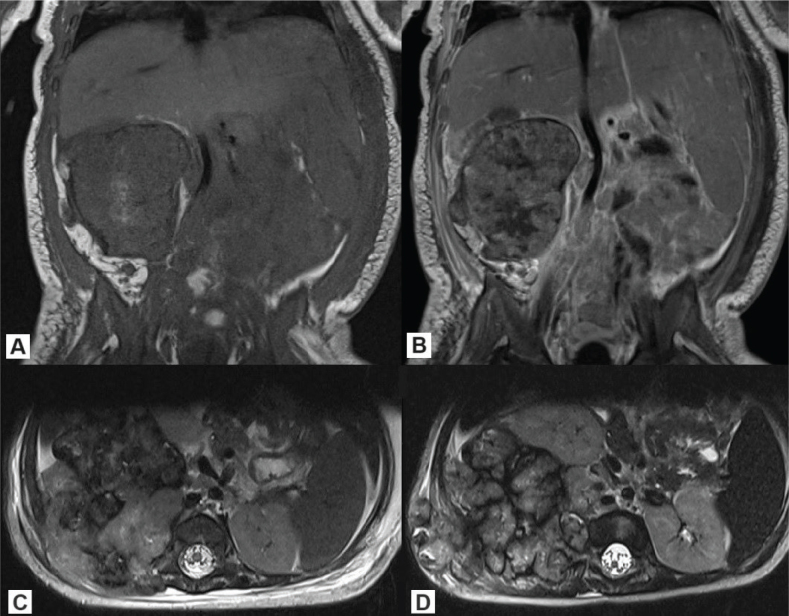
Lumbosacral MRI of case #1: (A) T1 sequence (coronal), (B) T1 contrast enhanced (coronal), (C) T2 sequence (axial) – at the moment of diagnosis; (D) T2 sequence (axial) at disease progression. MRI: Magnetic Resonance Imaging.

A previously healthy 3-month-old girl was evaluated for a hard and immovable lump lesion on the right lumbar region of her back. An abdomen Computed Tomography (CT) and a lumbosacral Magnetic Resonance Imaging (MRI) were performed and showed a mass of 7.4 × 6.1 × 8.0 cm in the right retroperitoneum with intrusion into the intravertebral foramen Th12-L4 exerting mass effect on the right kidney, liver, and right iliopsoas muscle. According to the Cooperative Weichteilsarkom Studiengruppe (CWS) Guidance 2014 Protocol, the patient was classified to the high risk group, of any histology, N1, IRS Group III, of any initial tumor size. MRI also showed bilateral abdominal and retroperitoneal lymphadenopathy (Figure 1). A biopsy was taken and immunohistochemical staining revealed CD99, FLI1, WT1, BCL-2 and CD31. Moreover, the tumor was partly positive for S-100P, CD56, CD68 and LCA, while negative for chromogranin A, synaptophysin, Myo D1, CD34, calretinin and BCOR. Final histopathological results confirmed the diagnosis of undifferentiated/unclassified small cell sarcoma. The metastatic workup using CT and positron emission tomography (PET) scan did not reveal any distant metastases. The patient received neo-adjuvant CWS-based chemotherapy composed of ifosfamide, vincristine and actinomycin-D. The patient was first treated with 50% chemotherapy dose reduction; however, as the treatment was well tolerated, the doses were escalated to full dose. After the primary tumor regression up to 6.3 × 3.9 × 3.8 cm after the first course of treatment, at 7th week of therapy, a local progression was diagnosed as well as local nodal and distant pulmonary metastases. The patient received the second line chemotherapy based on carboplatin and etoposide, and salvage, off-label therapy with combined irinotecan and temozolomide, but unfortunately the disease progressed despite both therapies. The patient died with malignancy progression a few weeks later.

### Case #2

**Figure 2:**
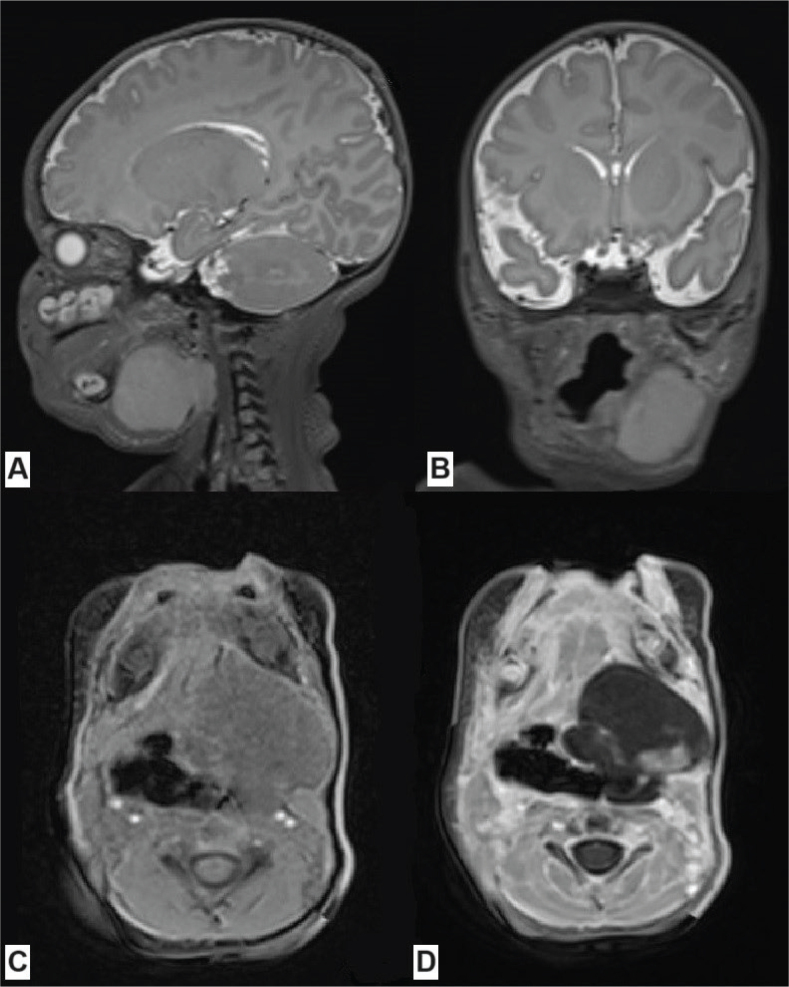
Craniofacial MRI of patient #2 at the time of diagnosis showing the tumor in the left submandibular region, modeling and displacing the left salivary gland, the styloglossus muscle, the posterior belly of the digastric muscle, the root of the tongue, the muscles of the mouth floor, the oral and laryngeal parts of the pharynx, and downwardly reaching the level of the middle part of the larynx: (A) T2 SPACE sequence (sagittal), (B) T2 SPACE sequence (coronal), (C) T1 VIBE sequence (axial), (D) T1 VIBE contrast enhanced sequence (axial). MRI: Magnetic Resonance Imaging.

A 3-week-old infant presented with a tumor on the left submandibular region. The baby was born healthy and the pregnancy had been uncomplicated. He did not have a family history of cancer. The tumor caused asymmetrical positioning of the tongue and distorted the palate. When turning his head to the right, the boy presented upper-airway dyspnea. A craniofacial MRI showed a polycyclic, irregular mass of 3.6 × 3.4 × 3.0 cm in the left submandibular region extending to the oropharynx and to the supraglottis (Figure 2). Abdominal and thoracic CT and PET scans did not show any distant metastases. According to the CWS Guidance 2014 Protocol, the patient was classified to the standard risk group, of any histology, N0, IRS Group III, of ≤ 5 cm (however, due to the high-grade tumor, the patient was stratified and treated as the high-risk group). A biopsy was taken, and histopathological examination showed large cells with bright, granular cytoplasm. Immunohistochemistry showed the tumor to be negative for CK AE1/AE3, EMA, ALK1, CD20, CD30, glipican3, CD117, LCA, PLAP, inhibin, desmin, INI1, MelanA, SOX10, S100, synaptophysin, GFAP and CD34, while positive for SATB2, BCL6 corepressor (BCOR), ERG, CD99 and FLI1. Due to the unclear histopathology results, a genetic study using the FusionPlex Sarcoma v2 panel from tumor DNA was performed. In the meantime, a tracheostomy was performed as the tumor was obstructing the airways. As the tumor progressed, vincristine and cyclophosphamide-based chemotherapy was started, although the final histological diagnosis was still lacking. Taking into account the clinical presentation of the tumor and the child’s age, a decision was made to implement chemotherapy for soft tissue rhabdoid tumors according to Eu-Rhab regimen with good tolerance. A day after the third course of chemotherapy using vincristine, anemia and painless jaundice developed. Laboratory findings showed anemia grade 3 and thrombocytopenia grade 4 as well as grade 4 increase of serum bilirubin levels and transaminases, according to Common Terminology Criteria for Adverse Events (CTCAE). Hepatitis serology showed negative results for HBsAg, antiHBs, and antiHBc, as well as CMV-DNA and EBV-DNA were negative. In the course of differential diagnostics hemolysis, hemophagoctic lymphohistiocytosis and other conditions were ruled out, and finally fulminant toxic liver failure, probably induced by vincristine, was diagnosed. Despite hepatoprotective and empirical antibiotic treatment, liver failure progressed rapidly, and hepatic encephalopathy developed. Because of further deterioration of general condition mainly related to hepatic and respiratory failure, the patient was transferred to the pediatric intensive care unit (PICU). Further significant increases in aminotransferases and bilirubin, as well as abnormalities in the coagulogram, were observed. Due to kidney failure, renal replacement therapy was implemented. Nevertheless, in the following days, the patient’s condition continued to deteriorate and he deceased on the 7th day after PICU admission. NGS results obtained after the patient’s death revealed undifferentiated sarcoma with BCOR-internal tandem duplication (BCOR-ITD).

## Discussion

The diagnosis of STUS causes many difficulties for clinicians. According to the 2020 WHO Classification of Tumors of Soft Tissue and Bone, the diagnosis is established mainly based on genetic criteria, without considering immunohistochemical ones. This is associated with a longer diagnostic delay, and thus causes a risk of local progression and distant metastasis. Due to the aggressive character of these tumors, this often necessitates the administration of chemotherapy before a final diagnosis is established [2].

One of the subtypes of STUS is BCOR-rearranged sarcoma that can be characterized with, e.g. BCOR::CCNB3 fusion [6] or BCOR-ITD [7]. This subtype is also present in pediatric patients, like our infant patient. The potential localizations are soft tissue of the trunk, abdomen, head and neck, and bones [8–10]. This mutation plays the role of a major oncogenic driver [11, 12] and is usually not associated with familial cancer predisposition syndromes [11]. It can be also present in pediatric clear cell sarcomas of the kidney [11, 12], central nervous system high-grade neuroepithelial tumors with BCOR alteration [13], primitive myxoid mesenchymal tumors of infancy [14], and high-grade uterine sarcoma [15]. According to Sparber-Sauer et al., primary tumors’ size is frequently bigger than 5 cm. Especially BCOR-rearranged sarcoma patients older than 13 years tend to have better 3-year event free survival (41.2%) [9].

The crucial part of local management of STUS is surgical treatment [16]; however, that is an option only if the location of the tumor allows resection. Moreover, most patients with initial unresected or unresectable tumors could benefit from a R0‐R1 delayed resection. Pediatric patients with BCOR-ITD sarcomas that were free of disease only in case of radical surgical treatment with IVA (ifosfamide-vincristine-actinomycin-D) adjuvant chemotherapy or VACA (vincristine-actinomycin-D-cyclophosphamide-adriamycin) neoadjuvant chemotherapy with radical residual mass resection [4, 17, 18]. Unfortunately, in both of our patients, radical surgical treatment was impossible. Surgery should be followed by further systemic treatment which can be fatally toxic [19] and Ogun et al. [5]. The lack of clear chemotherapy guidelines for the treatment of STUS makes impossible to apply the optimal treatment implementation of this aggressive, rapidly progressive pediatric tumor subtype. The use of doxorubicin in the first line [20], protocols for Ewing’s sarcoma [21], the ICE (ifosfamide-carboplatin-etoposide) reinducing regimen [22], IVA plus carboplatin, epirubicin and etoposide [23] was studied. However, their efficacy remains equivocal.

Due to the higher water content of neonates and thus the increased volume of distribution of hydrophilic drugs, achieving therapeutic concentrations is more difficult. On the contrary, due to the increased adipose tissue content, excessive toxicity of lipophilic drugs may be observed. An analogous effect may be observed due to lower albumin concentrations leading to a higher free fraction of drugs, especially those that bind strongly to transport proteins. Because of the immaturity of some hepatic enzyme systems, including the impaired coupling of drug metabolites to glucuronic acid or glycine and impaired acetylation processes, the time of drug metabolism and thus their half-life may be prolonged. The shedding of drugs amid reduced hepatic and renal clearance in the face of poorer renal blood supply and glomerular and tubular function compared with older children means that toxic metabolites may lead to increased adverse effects, such as hepatotoxicity, also life-threatening after the use of some cytostatic drugs [24–29]. Probably this was an additional reason for liver failure in our patient.

Radiotherapy cannot be administered to infants. The role of radiotherapy has declined over the past decades, because of the increasing knowledge of high susceptibility of immature tissues to irradiation, even if its usage could result in tumor control. Late effects include growth failure due to the damage caused to growth cartilages, neurocognitive impairment, endocrinopathies, and subsequent neoplasms.

Both of our cases depict the difficulties with the proper adjustment of systemic treatment in primary unresectable tumors in infants [30]. Major symptoms resulted primarily from the unfavorable localization and size of the tumor. The first patient presenting retroperitoneal undifferentiated small cell round sarcoma with typically reduced CD56 expression [31] was administered standard chemotherapy for RMS-like sarcomas. Chemotherapy was well tolerated. However, rapid progression was observed. The other patient presented sarcoma with BCOR-ITD of the undescribed length of duplication [14], with typical BCOR and SATB2 expression [32]. He was treated with vincristine, cyclophosphamide, and doxorubicin in anticipation of the histopathological report, due to the emergency based on the clinical course of the disease. It led to severe toxicities, mainly hepatic, that finally contributed to the fatal outcome. The comparison of chemotherapy in both infantile patients emphasizes the difficult match of chemotherapeutics applied in infantile highly aggressive solid tumors, taking into consideration both adequate response to treatment and life-threatening treatment toxicities.

In conclusion, the cases presented are examples of infantile undifferentiated sarcomas that pose great diagnostic and therapeutic difficulties. A full molecular assessment is needed when a diagnosis of STUS is suspected. The main therapeutic problem is the need to inflate the intensity of systemic oncological treatment to eliminate the intensely dividing sarcoma cells and its potential toxicities for immature infant organs. Collecting data on rare infantile undifferentiated sarcomas is crucial to optimize and standardize treatment, and to improve prognosis. Moreover, prospective studies are needed to optimize the treatment strategy of STUS. Concerning the poor survival related to standard therapies, novel treatment options, especially targeted therapies, are desperately needed [33].

## Data Availability

Data presented in the paper are available upon reasonable request.
